# Functional Differences between E. coli and ESKAPE Pathogen GroES/GroEL

**DOI:** 10.1128/mBio.02167-20

**Published:** 2021-01-12

**Authors:** Jared Sivinski, Andrew J. Ambrose, Iliya Panfilenko, Christopher J. Zerio, Jason M. Machulis, Niloufar Mollasalehi, Lynn K. Kaneko, Mckayla Stevens, Anne-Marie Ray, Yangshin Park, Chunxiang Wu, Quyen Q. Hoang, Steven M. Johnson, Eli Chapman

**Affiliations:** a Department of Pharmacology and Toxicology, College of Pharmacy, University of Arizona, Tucson, Arizona, USA; b Department of Chemistry and Biochemistry, University of Arizona, Tucson, Arizona, USA; c Center for Innovation in Brain Science, Tucson, Arizona, USA; d Department of Pharmacology, College of Medicine, University of Arizona, Tucson, Arizona, USA; e Department of Biochemistry and Molecular Biology, Indiana University School of Medicine, Indianapolis, Indiana, USA; f Stark Neurosciences Research Institute, Indiana University School of Medicine, Indianapolis, Indiana, USA; g Department of Neurology, Indiana University School of Medicine, Indianapolis, Indiana, USA; Louis Stokes Veterans Affairs Medical Center

**Keywords:** antibiotic, antimicrobial, chaperone, chaperonin, ESKAPE, GroEL, GroES, HSP10, HSP60

## Abstract

As the GroES/GroEL chaperonin system is the only bacterial chaperone that is essential under all conditions, we have been interested in the development of GroES/GroEL inhibitors as potential antibiotics. Using Escherichia coli GroES/GroEL as a surrogate, we have discovered several classes of GroES/GroEL inhibitors that show potent antibacterial activity against both Gram-positive and Gram-negative bacteria. However, it remains unknown if E. coli GroES/GroEL is functionally identical to other GroES/GroEL chaperonins and hence if our inhibitors will function against other chaperonins. Herein we report our initial efforts to characterize the GroES/GroEL chaperonins from clinically significant ESKAPE pathogens (Enterococcus faecium, Staphylococcus aureus, Klebsiella pneumoniae, Acinetobacter baumannii, Pseudomonas aeruginosa, and *Enterobacter* species). We used complementation experiments in GroES/GroEL-deficient and -null E. coli strains to report on exogenous ESKAPE chaperone function. In GroES/GroEL-deficient (but not knocked-out) E. coli, we found that only a subset of the ESKAPE GroES/GroEL chaperone systems could complement to produce a viable organism. Surprisingly, GroES/GroEL chaperone systems from two of the ESKAPE pathogens were found to complement in E. coli, but only in the strict absence of either E. coli GroEL (P. aeruginosa) or both E. coli GroES and GroEL (E. faecium). In addition, GroES/GroEL from S. aureus was unable to complement E. coli GroES/GroEL under all conditions. The resulting viable strains, in which E. coli *groESL* was replaced with ESKAPE *groESL*, demonstrated similar growth kinetics to wild-type E. coli, but displayed an elongated phenotype (potentially indicating compromised GroEL function) at some temperatures. These results suggest functional differences between GroES/GroEL chaperonins despite high conservation of amino acid identity.

## Abstract (precis)

The GroES/GroEL chaperonin from E. coli has long served as the model system for other chaperonins. This assumption seemed valid because of the high conservation between the chaperonins.

## INTRODUCTION

The GroES/GroEL chaperonin system is a nearly megadalton molecular machine that acts as an “Anfinsen cage” in the bacterial cytosol. This cage is formed when GroES interacts with GroEL to form a privileged hydrophilic chamber, where newly synthesized polypeptides or stress-denatured proteins can fold in an isolated chamber sequestered from the crowded cellular environment. The ∼800-kDa bis-toroidal tetradecamer GroEL binds unfolded polypeptides and, in an ATP-dependent manner, binds the ∼70-kDa heptameric GroES cochaperone, which acts as a lid to cap off the GroEL/GroES folding chamber (1–5). After ∼5 to 10 s, ATP is hydrolyzed, which allows for the disassembly of the Anfinsen cage and advances the catalytic cycle. More than 300 different proteins have been shown to interact with GroEL in Escherichia coli, ∼50 of which have been shown to completely rely on this chaperone system for proper folding (6, 7). Because some of these client proteins are essential, the GroES/GroEL chaperonin is essential for growth under all conditions (8). Based on conserved sequence and structure, it is thought that the homologous GroES/GroEL chaperonins from all organisms should be essential, although this has not been rigorously demonstrated (9, 10). The essential nature of GroES/GroEL and its clients led us to hypothesize that targeting these chaperonin systems might be an effective antibiotic strategy. Supporting this hypothesis, we previously developed a high-throughput screen (11) and identified several inhibitor scaffolds that are effective against bacteria (12–15) and trypanosomes (16); however, we have yet to definitively show that the compounds function by targeting the chaperonins.

**FIG 1:**
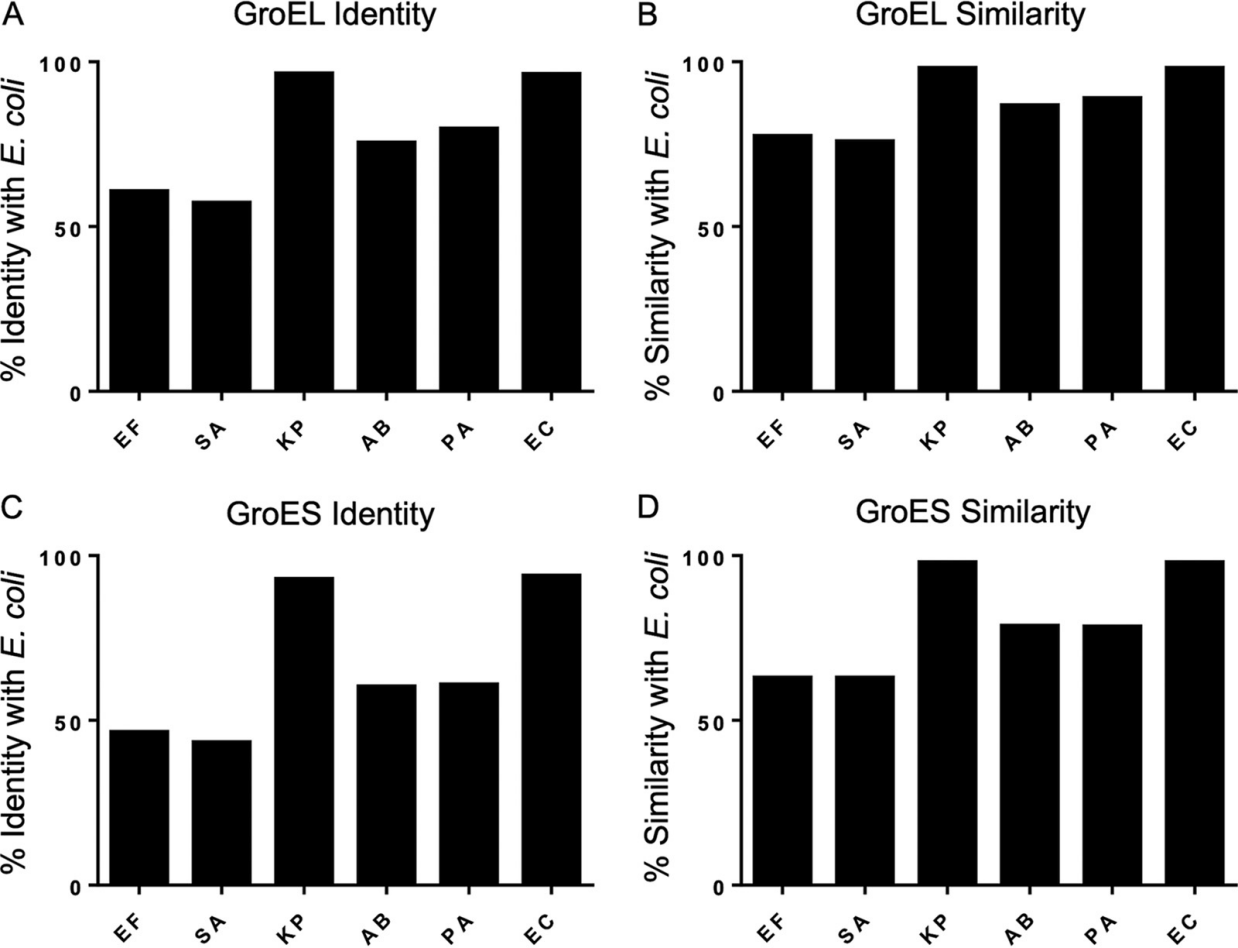
E. coli GroES/GroEL shares high amino acid identity and similarity with ESKAPE pathogens GroES/GroEL. Percentages of GroES/GroEL protein identity and similarity were generated from EMBOSS Needle protein alignment of E. coli GroES/GroEL and ESKAPE pathogen GroESL. (A) E. coli GroEL protein identity compared to ESKAPE GroEL. (B) E. coli GroEL protein similarity compared to ESKAPE GroEL. (C) E. coli GroES protein identity compared to ESKAPE GroES. (D) E. coli GroES protein similarity compared to ESKAPE GroES. EF, E. faecium; SA, S. aureus; KP, K. pneumoniae; AB, A. baumannii; PA, P. aeruginosa; EC, E. cloacae.

Taking advantage of the essential nature of GroES/GroEL in E. coli, in the present study, we conducted a series of genetic complementation experiments to study chaperonins from a panel of bacteria known as the ESKAPE pathogens (“ESKAPE” being an acronym for the Gram-positive bacteria Enterococcus faecium and Staphylococcus aureus and Gram-negative bacteria Klebsiella pneumoniae, Acinetobacter baumannii, Pseudomonas aeruginosa, and *Enterobacter* species) (17). We have been investigating the ESKAPE pathogens due in part to their broad range of evolutionary distances from E. coli and in particular because of their clinical relevance to human disease. The GroEL chaperonins from the ESKAPE pathogens are all greater than 50% identical (Fig. 1A) and 70% similar (Fig. 1B; see Table S1 in the supplemental material) to that of E. coli. In addition, although GroES chaperonins are not as highly conserved, all of them are greater than 40% identical and 60% similar (Fig. 1C and D; Table S1). Therefore, we hypothesized that GroES/GroEL from the ESKAPE pathogens would efficiently rescue a GroES/GroEL-deficient strain of E. coli, called LG6 (18). As in E. coli, only a single copy of *groESL* exists within the genomes of the ESKAPE pathogens, and where tested, *groESL* was found to be essential in the ESKAPE organisms (19–23). Surprisingly, we found that not all the ESKAPE chaperonins rescued GroES/GroEL-deficient E. coli, with some forming a dominant-negative phenotype. We questioned whether coexpression of endogenous E. coli GroES/GroEL (ESL^Coli^) with the plasmid-containing ESKAPE GroES/GroEL (ESL^ESKAPE^) could generate mixed-heptameric or tetradecamer chaperones with compromised function. To explore our hypothesis, a stepwise strategy was used starting from GroES/GroEL-deficient E. coli, moving next to *groEL*-null E. coli, and then finally moving to full replacement of E. coli *groESL* with ESKAPE *groESL*. Ultimately, we show that the function of ESKAPE GroES/GroEL in E. coli is dictated by the activity of GroESL^Coli^-GroESL^ESKAPE^ hetero-oligomeric complexes, demonstrating functional divergence among the highly conserved chaperonins.

10.1128/mBio.02167-20.2 TABLE S1 E. coli shares high amino acid similarity with ESKAPE pathogens^a^. ^a^GroESL protein similarity (%) generated from EMBOSS Needle protein alignment of E. coli GroESL and ESKAPE pathogens. (A) GroESL protein similarity (%). (B) GroES protein similarity (%). (C) GroEL protein similarity (%). Color gradient demonstrates highest similarity highlighted in red and lowest similarity in white. Download Table S1, PDF file, 0.1 MB. Copyright © 2021 Sivinski et al. 2021 Sivinski et al. This content is distributed under the terms of the Creative Commons Attribution 4.0 International license.

## RESULTS

### E. coli GroES/GroEL shares high amino acid identity and similarity with ESKAPE pathogen GroES/GroEL.

Although structural information has yet to be generated for the ESKAPE GroES/GroEL oligomers, alignment of their primary sequences with E. coli MG1655 K-12 GroES/GroEL argues for conserved structure and function (Fig. 1; see Table S1, Table S2, and Fig. S1 in the supplemental material). Amino acid identity is higher between the Gram-negative pathogens and E. coli (60.2 to 93.8% for GroES and 75.4 to 96.4% for GroEL) compared to Gram-positive pathogens and E. coli (43.3 to 46.4% for GroES and 57.1 to 60.6% for GroEL) (Fig. 1A and C). This trend is also observed for amino acid similarity in the Gram-negative bacteria (78.4 to 97.9% for GroES and 86.7 to 98.0% for GroEL) and Gram-positive bacteria (62.9% for GroES and 75.8 to 77.4% for GroEL) (Fig. 1B and D). Furthermore, predicted ESKAPE GroES and GroEL isoelectric points (4.87 to 5.38 and 4.56 to 5.04, respectively) are congruent with those of E. coli GroES and GroEL (5.15 and 4.85, respectively) (Table S2). The isoelectric points of the Gram-positive ESKAPE pathogens are least like those of E. coli. Overall, E. faecium and S. aureus GroES/GroEL contain fewer residues than E. coli GroES/GroEL and lack the GGM C-terminal repeat found in E. coli GroES/GroEL (24) and other Gram-negative ESKAPE pathogens.

10.1128/mBio.02167-20.1 FIG S1 Comparisons of the GroEL sequences from E. coli and the ESKAPE pathogens. The respective panels show GroEL sequence comparisons between E. coli 4pko.1.B with E. faecium (EF), S. aureus (SA), K. pneumoniae (KP), A. baumannii (AB), P. aeruginosa (PA), and E. cloacae (EC). Identical residues are shown in green, similar residues are shown in purple, and different residues are shown in red. Download FIG S1, PDF file, 0.4 MB. Copyright © 2021 Sivinski et al. 2021 Sivinski et al. This content is distributed under the terms of the Creative Commons Attribution 4.0 International license.

10.1128/mBio.02167-20.3 TABLE S2 ESKAPE pathogen GroES/GroEL is predicted to be structurally similar with comparable net charge in their respective cellular environments compared to MG1655 GroES/GroEL^a^. ^a^Isoelectric point and molecular weight data were generated using the ExPASy Computer pI/MW data tool. Gram-positive bacteria (E. faecium and S. aureus) have overall fewer residues and a lower isoelectric point and lack the C-terminal GGM repeat compared to Gram-negative bacteria (*K. pneumonia*, A. baumannii, P. aeruginosa, E. cloacae, and E. coli). Download Table S2, PDF file, 0.05 MB. Copyright © 2021 Sivinski et al. 2021 Sivinski et al. This content is distributed under the terms of the Creative Commons Attribution 4.0 International license.

### Only K. pneumoniae, A. baumannii, and E. cloacae GroES/GroEL rescue GroES/GroEL-deficient E. coli.

**FIG 2:**
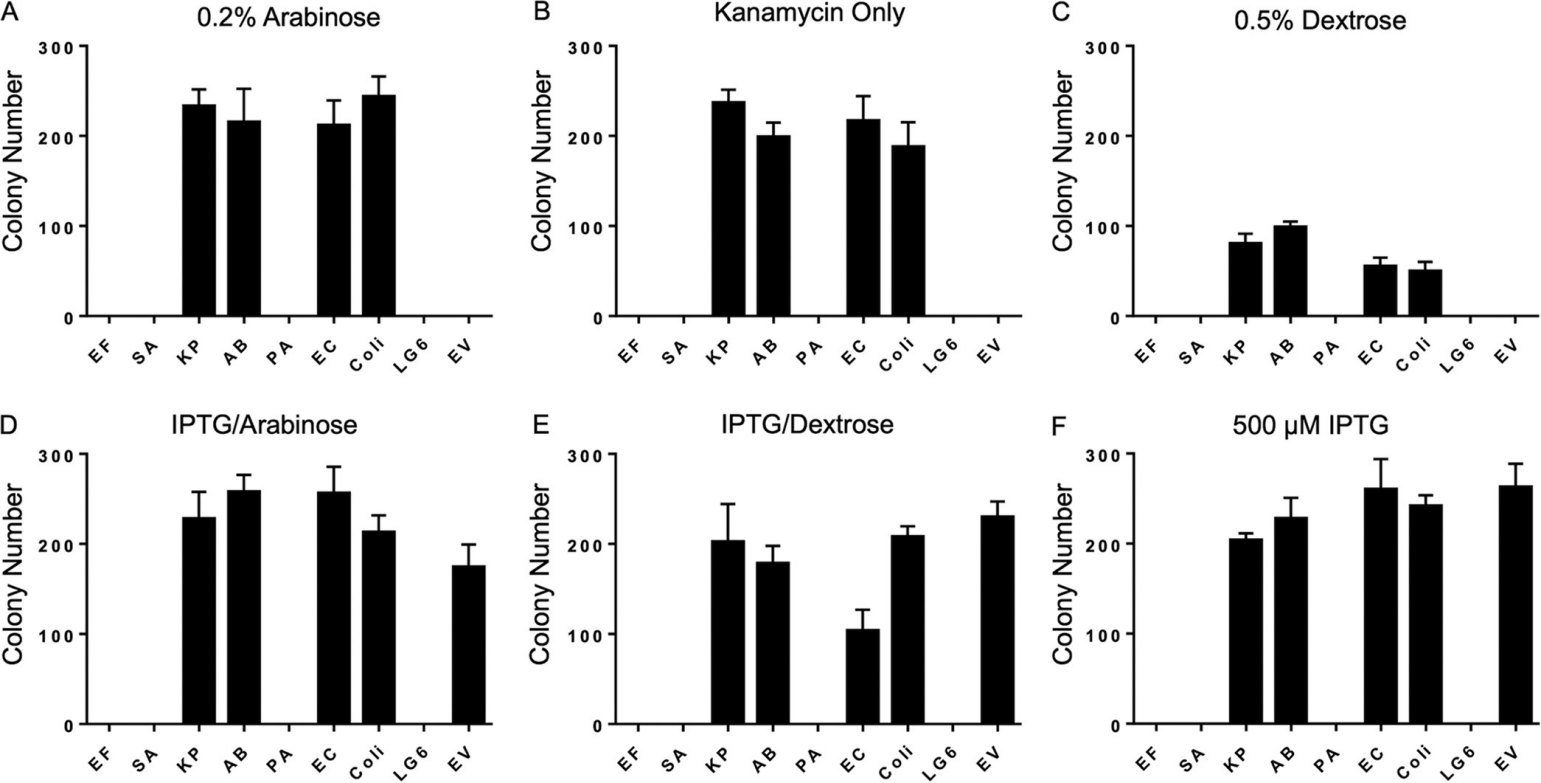
Only K. pneumoniae, A. baumannii, and E. cloacae GroES/GroEL rescue GroES/GroEL-deficient E. coli. Shown is the LG6 colony number from antibiotic selection plate reported after transformation with individual ESKAPE *pBAD*-promoted *groESL* (Km^r^) plasmid, E. coli *pBAD groESL* (Km^r^) plasmid, or *pBAD* (Km^r^) empty vector. LG6 (Cm^r^) did not grow on kanamycin plates. (A) With 0.2% arabinose/kanamycin. (B) With kanamycin only. (C) With 0.5% dextrose–kanamycin. (D) With 500 μM IPTG–0.2% arabinose–kanamycin. (E) With 500 μM IPTG–0.5% dextrose–kanamycin. (F) With 500 μM IPTG–kanamycin. Data represent at least three independent experiments and are reported as mean with standard deviation (SD). EF, E. faecium; SA, S. aureus; KP, K. pneumoniae; AB, A. baumannii; PA, P. aeruginosa; EC, E. cloacae; Coli, E. coli; EV, empty vector.

Plasmids with *pBAD*-driven ESKAPE *groESL* were used to determine if GroES/GroEL from the ESKAPE pathogens could complement a GroES/GroEL-deficient E. coli cell line, LG6 (18). LG6 contains a *lac*-promoted *groESL* operon that, in the absence of lactose or IPTG (isopropyl-β-d-thiogalactopyranoside) fails to produce sufficient endogenous GroES/GroEL to sustain viable colonies (25). When LG6 cells were transformed with a plasmid containing *pBAD*-driven *groESL* from the ESKAPE pathogens or E. coli (as a positive control) and induced with arabinose, E. faecium, S. aureus, and P. aeruginosa GroES/GroEL chaperone systems could not rescue GroES/GroEL-deficient LG6 cells (Fig. 2A). This was also the case for untransformed LG6 and LG6 transformed with *pBAD*-driven empty vector (Fig. 2A). We reasoned this could be due to inappropriate protein levels and thus probed the system with lower levels of ESKAPE and E. coli GroES/GroEL. Plasmid antibiotic selection alone (without inducing agent) demonstrated that there was significant transcriptional leakage from the *pBAD* promoter of the *groESL* plasmids, and the same ESKAPE *groESL* plasmids rescued LG6 similarly as when induced with arabinose (compare Fig. 2A and B). Addition of dextrose to the agar plate, to suppress transcriptional leakage, reduced the number of viable colonies of K. pneumoniae, A. baumannii, and Enterobacter cloacae, but failed to produce any viable LG6 colonies in E. faecium, S. aureus, and P. aeruginosa *groESL*-transformed cells (Fig. 2C). Surprised by these results, we induced the chromosomal E. coli *groESL* operon to demonstrate that LG6 could still be rescued by GroESL^Coli^. Despite adequate levels of GroESL^Coli^, we found that in the presence of the ESKAPE *groESL* plasmids, E. faecium, S. aureus, and P. aeruginosa GroES/GroEL-containing cells were unable to produce viable organisms. However, empty vector, K. pneumoniae, A. baumannii, E. cloacae, and E. coli *groESL* plasmid-containing LG6 cells were viable in the presence of wild-type, chromosomal GroES/GroEL stimulated by IPTG induction (Fig. 2D, E, and F).

### All *pBAD groESL* plasmids from Gram-negative KAPE pathogens rescued transformed AI90 cells after the *sacB* pACYC E. coli *groESL* plasmid was counterselected.

**FIG 3:**
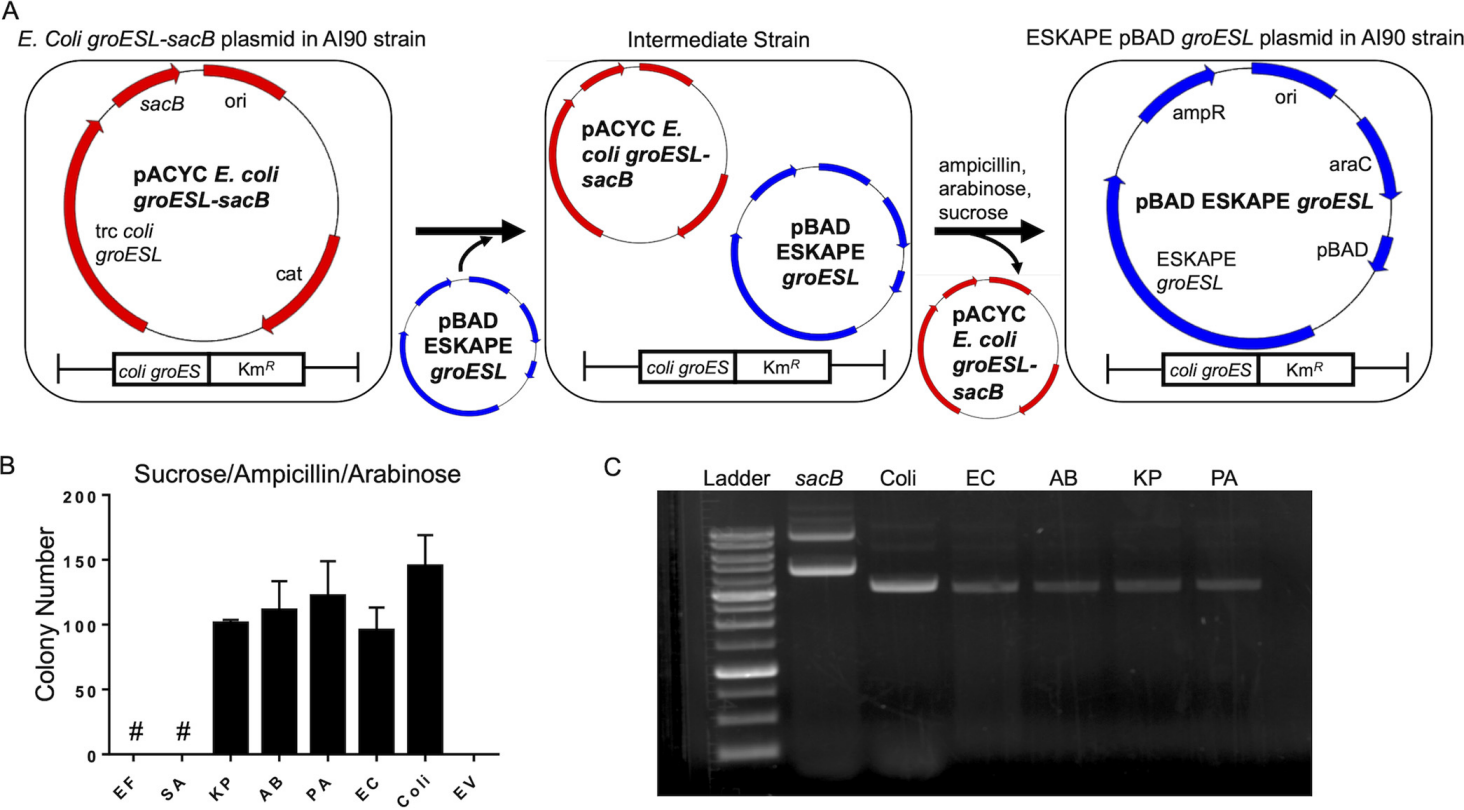
All *pBAD groESL* plasmids from Gram-negative ESKAPE pathogens rescue transformed AI90 after the *sacB* pACYC E. coli *groESL* plasmid is counterselected. (A) Scheme of ESKAPE *groESL* plasmid shuffle into the E. coli *groEL*-null background AI90 strain. (B) AI90 colony number from 5% sucrose–0.2% arabinose–ampicillin selection plate reported after transformation with individual ESKAPE *pBAD groESL* (Amp^r^) plasmid, E. coli pBAD *groESL* (Amp^r^) plasmid, or *pBAD* (Amp^r^) empty vector. The symbol “#” indicates colonies were visualized on these plates but retained mutant *sacB groEL* plasmid. Results represent three independent experiments and are reported as mean with SD. (C) All Gram-negative ESKAPE pathogens rescued *groEL*-deficient AI90 after *sacB* pACYC *groEL* (Cm^r^) plasmid shuffle. Plasmids from surviving colonies after shuffle were isolated and run on 0.5% DNA gel. Ladder, DNA ladder; *sacB*, *sacB* pACYC E. coli *groESL* plasmid; Coli, *pBAD* E. coli *groESL*; EC, *pBAD* E. cloacae *groESL*; AB, *pBAD* A. baumannii *groESL*; KP, *pBAD* K. pneumoniae *groESL* plasmid; PA, *pBAD* P. aeruginosa *groESL* plasmid.

After ruling out gene dosage (Fig. 2) and codon bias (Table S3) as factors that prevented E. faecium, S. aureus, and P. aeruginosa *groESL* from rescuing LG6 cells, we sought to determine the cause of these observed dominant-negative phenotypes. To rule out ring mixing between GroEL^Coli^ and GroEL^ESKAPE^ as the cause of the dominant-negative effect, we expressed ESKAPE GroES/GroEL in AI90 E. coli cells, in which *groES* is present, but *groEL* is absent from the chromosome. AI90 is maintained by an E. coli *groESL* plasmid capable of negative selection due to the presence of *sacB* within this pACYC construct (7). AI90 cells were transformed with ESKAPE *groESL* plasmids in the presence of sucrose (negative pACYC E. coli *groESL sacB* selection) and kanamycin (positive ESKAPE *groESL* selection). This selection shuffled out the E. coli *groESL* plasmid, forcing reliance on the ESKAPE *groESL* plasmids for survival. This platform eliminated the possibility of forming mixed-GroEL tetradecamers (active or inactive) and more conclusively tested the compatibility of ESKAPE GroES/GroEL in E. coli (Fig. 3A). We found that all ESKAPE pathogen GroELs that could complement LG6 also rescued E. coli *groEL*-null AI90 (Fig. 3B). Because *sacB* is subject to mutations that render its gene product unable to kill cells that retain this plasmid (26), postselection colonies were amplified and plasmid DNA purified to confirm the absence of the pACYC *groESL* plasmids and the presence of an ESKAPE *groESL* plasmid (Fig. 3C). P. aeruginosa GroES/GroEL was able to complement AI90, but not LG6, supporting our previous observation that P. aeruginosa GroEL formed inactive/underactive mixed-GroEL rings in the presence of E. coli GroEL (Fig. 2) and is responsible for the dominant-negative effect seen in LG6. This observation could not explain the lack of AI90 E. coli rescue in the presence of E. faecium or S. aureus GroEL, although E. faecium GroEL may be incompatible with E. coli GroES in this system.

10.1128/mBio.02167-20.4 TABLE S3 ESKAPE pathogen GroES/GroEL require less than 3% E. coli rare codons. CAIcal codon usage per 1,000 bp of ESKAPE and E. coli *groESL*. E. coli rare codons are highlighted in red, with relative codon usage per 1,000 bp quantified in blue. EF, E. faecium; SA, S. aureus; KP, K. pneumoniae; AB, A. baumannii; PA, P. aeruginosa; EC, E. cloacae; Coli, E. coli. Download Table S3, PDF file, 0.3 MB. Copyright © 2021 Sivinski et al. 2021 Sivinski et al. This content is distributed under the terms of the Creative Commons Attribution 4.0 International license.

### Viable ESKAPE *groESL* knock-ins were generated by λ-red recombineering in MG1655.

**FIG 4:**
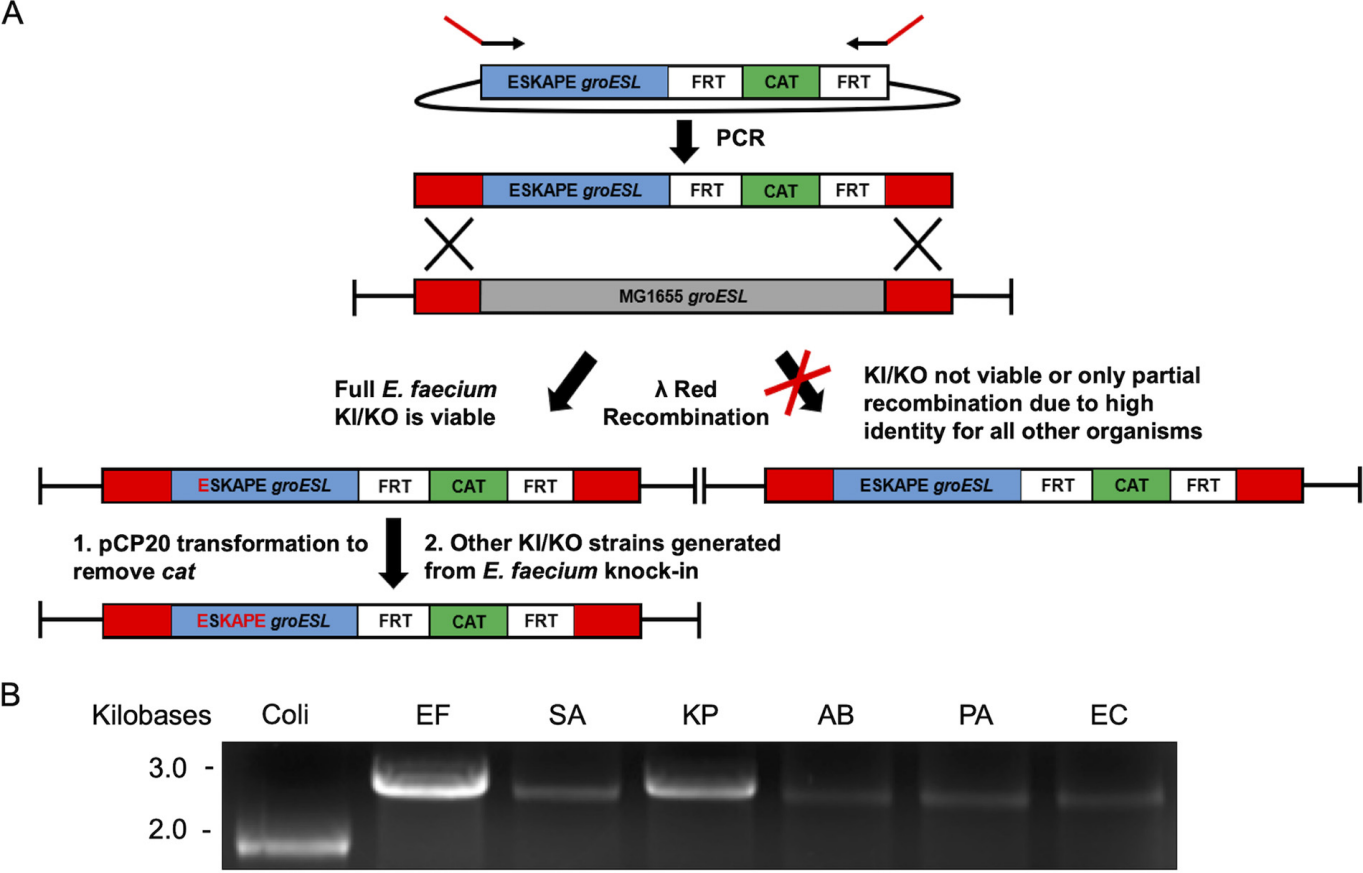
Viable ESKAPE *groESL* knock-ins were generated by λ-red recombineering in MG1655. (A) Due to high sequence identity between ESKAPE pathogens and E. coli *groESL*, only MGΔ*groESL*::EF *groESL* (Cam^r^) could be obtained from knock-in (lower *groESL* sequence homology compared to Gram-negative pathogens). From this knock-in, K. pneumoniae, A. baumannii, P. aeruginosa, and E. cloacae *groESL* knock-ins were generated. Full S. aureus *groESL* knock-in could not be obtained. (B) PCR products for MG1655 and knock-ins for all ESKAPE pathogens using primers flanking the *groESL* gene visualized on agarose gel. Coli, E. coli MG1655 WT *groESL*; EF, MGΔ*groESL*::EF *groESL* (Cam^r^); SA, MGΔ*groESL*::SA *groESL* (Cam^r^) partial knock-in; KP, MGΔ*groESL*::KP *groESL* (Cam^r^); AB, MGΔ*groESL*::AB *groESL* (Cam^r^); PA, MGΔ*groESL*::PA *groESL* (Cam^r^); EC, MGΔ*groESL*::EC *groESL* (Cam^r^).

Because AI90 contains *groES* on the chromosome, we sought to eliminate the possibility of generating inactive GroESL^Coli^-GroESL^ESKAPE^ mixed complexes within the E. coli chaperonin complex. To accomplish this, E. coli *groESL* was replaced by ESKAPE *groESL* using λ-red recombination (27, 28) (Fig. 4A). Because of the high base pair identity between the Gram-negative ESKAPE pathogen and E. coli *groESL*, complete knock-ins for these organisms could not initially be generated. Lower base pair homology between E. faecium and E. coli was sufficient for complete knock-in of E. faecium *groESL* into the E. coli *groESL* operon. The E. faecium *groESL* strain was then used as a template to knock in *groESL* from the remaining ESKAPE pathogens, and each successful knock-in cell line was confirmed by sequencing. In the absence of background E. coli GroES or GroEL, we discovered that all but one of the ESKAPE pathogen GroES/GroEL chaperone systems could replace E. coli *groESL* in MG1655 (Fig. 4B). The ability of E. faecium to rescue in this context, but not in AI90, indicates that GroES ring mixing between E. faecium and E. coli may produce a dominate-negative phenotype. It should not be discounted that GroES^Coli^ and GroEL^E. faecium^ may not interact or may form a trapped complex incapable of refolding clients. Complete S. aureus *groESL* knock-in was not possible using this system, but we have observed that S. aureus GroES/GroEL forms inclusion bodies when expressed in BL21 cells with host GroES/GroEL. The underlying biochemical reason for this remains unknown and is under investigation.

### Coexpression of GroEL^ESKAPE^ and E. coli GroEL^D473C/532Δ^ forms nonfunctional-tetradecameric GroEL hetero-oligomers.

**FIG 5:**
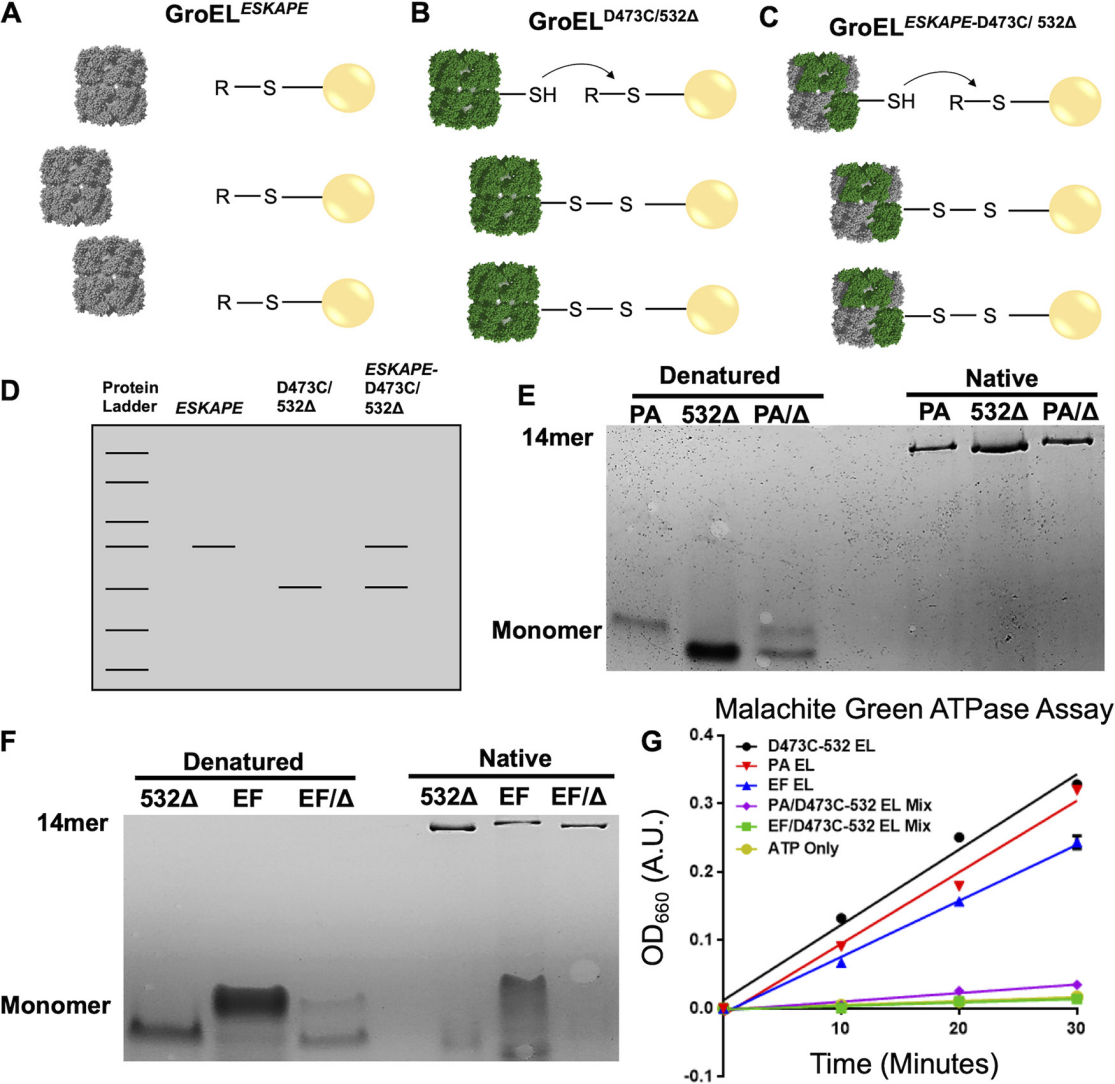
Coexpression of GroEL^ESKAPE^ and E. coli GroEL^D473C/532Δ^ forms nonfunctional-tetradecameric GroEL hetero-oligomers. (A) GroEL^ESKAPE^ was expressed in its respective knock-in strain (Fig. 4), purified by Q Sepharose FF (FFQ), and incubated with thiopropyl Sepharose (TPS) resin, but does not bind to resin. (B) GroEL^D473C/532Δ^ (cysteine and truncation mutant) was expressed in BL21, purified by FFQ, captured on TPS resin, and then eluted with increasing concentrations of DTT. (C) GroEL^ESKAPE^ was coexpressed with GroEL^D473C/532Δ^ in BL21, purified by FFQ, captured on TPS resin, and then eluted with increasing concentrations of DTT. (D) GroEL^D473C/532Δ^ runs at a lower molecular weight than GroEL^ESKAPE^ by SDS-PAGE. Captured hetero-oligomer DTT elution fractions, made up of GroEL^ESKAPE^ and GroEL^D473C/532Δ^ displays two bands, representing a mixed-GroEL ring. (E) Denatured (DTT, heat, and SDS treated) or nondenatured samples were run on a 4 to 10% native gradient gel and visualized by Coomassie brilliant blue staining. The fractions analyzed were non-DTT fraction GroEL^P. aeruginosa^ (PA), DTT fraction GroEL^D473C/532Δ^ (532Δ), and DTT fraction GroEL^P. aeruginosa^*^/^*^D473C/532Δ^ mixed complex (PA/Δ). (F) Denatured (DTT, heat, and SDS treated) or native samples were run on a 4 to 10% native gradient gel and visualized by Coomassie brilliant blue staining. The fractions analyzed were non-DTT fraction GroEL^E. faecium^ (EF), DTT fraction GroEL^D473C/532Δ^ (532Δ), and DTT fraction GroEL^E. faecium^*^/^*^D473C/532Δ^ mixed complex (EF/Δ). (G) Malachite green ATPase assay using 50 nM GroEL and 100 μM ATP measured at 660 nm over time. Black, GroEL^D473C/532Δ^; red, GroEL^P. aeruginosa^; blue, GroEL^E. faecium^; pink, GroEL^P. aeruginosa^*^/^*^D473C/532Δ^; green, GroEL^E. faecium^*^/^*^D473C/532Δ^; gold, ATP only (spontaneous ATP hydrolysis).

The genetic data from the AI90 rescue and λ-red recombineering experiments argued for the formation of mixed GroEL complexes, but we wanted to demonstrate mixed complex formation and to determine if these had compromised biochemical function. It has been previously shown that coexpression of GroEL^Coli^ and GroEL^Coli-mutant^ monomers form mixed GroEL tetradecamers with monomer integration directly correlated with the level of expression of each GroEL monomer type (29, 30). To investigate the formation of GroEL hetero-oligomers in E. coli, pBAD-driven GroEL^ESKAPE^ (expressed alone in its respective knock-in strain Fig. 4) (Fig. 5A), *lacIq-Ptac* GroEL^D473C/532Δ^ (expressed alone in BL21 E. coli) (Fig. 5B), or both were briefly coexpressed in BL21 (Fig. 5C). Overexpressed GroEL was first purified by ion exchange and then incubated with thiopropyl Sepharose (TPS) resin and allowed to air oxidize. It is expected that the GroEL cysteine mutant (D473C/532Δ) will form a covalent bond with the TPS resin and elute after reduction with dithiothreitol (DTT), whereas GroEL^ESKAPE^ will be found in the flowthrough and not in the DTT-eluted fractions because it lacks the reactive cysteine. Furthermore, to differentiate between GroEL^ESKAPE^ and GroEL^D473C/532Δ^, active truncated E. coli GroEL (532Δ mutant) was used to determine GroEL identity by SDS-PAGE (Fig. 5D). Coexpressed GroEL^D473C/532Δ^ and GroEL^P. aeruginosa^ protein eluted with DTT from TPS resin was found to be a tetradecamer by native PAGE (Fig. 5E). The DTT-eluted fraction species was present as a double band by PAGE under denaturing conditions, indicating the formation of hetero-oligomeric GroEL *in vivo*. (Fig. 5E). This experimental design was replicated using GroEL^D473C/532Δ^ and GroEL^E. faecium^ which yielded similar results (Fig. 5F). Importantly, these GroEL hetero-oligomers showed severely impaired ATPase activity (Fig. 5G) compared to purified homo-oligomers generated using strains from Fig. 4 or GroEL^D473C/532Δ^ expressed in BL21 alone.

### ESKAPE GroEL domain replacement by E. coli GroEL domains produces functional chimeras capable of rescuing GroES/GroEL-deficient E. coli.

**FIG 6:**
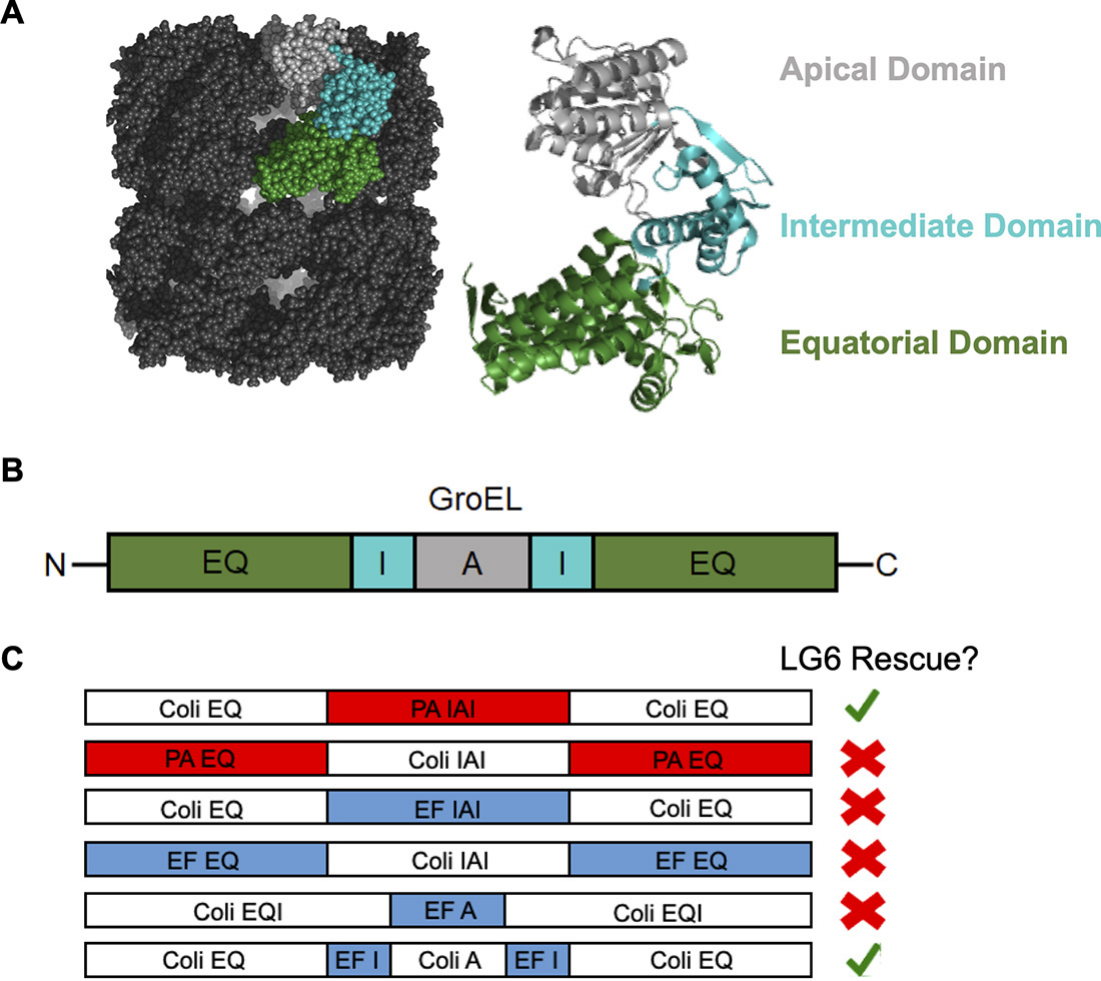
ESKAPE GroEL domain replacement by E. coli GroEL domains produces functional chimeras capable of rescuing GroES/GroEL-deficient E. coli. Chimeras were tested for their ability to rescue LG6 in cases where ESKAPE GroEL formed a dominant-negative phenotype. All plasmids contain E. coli *groES* upstream of the *groEL* chimera. (A) E. coli GroEL tetradecamers and monomer (PDB 1SX3) with labeled apical (gray), intermediate (teal), and equatorial (forest green) domains. (B) Outline of GroEL domains from N to C terminus. Equatorial (EQ; forest green), intermediate (I; teal), and apical (A; gray) domains. (C) Replacing the P. aeruginosa (PA) equatorial domain with the E. coli (Coli) equatorial domain and replacing the E. faecium (EF) equatorial and apical domains with E. coli equatorial and apical domains produced viable (green checkmark) LG6 colonies when these chimeras were expressed from *pBAD*-promoted plasmids. All other chimeras could not rescue LG6 (red X mark).

Based upon the E. faecium *and* P. aeruginosa GroES/GroEL dominant-negative phenotype in LG6 (Fig. 2) and the formation of GroEL^ESKAPE^ and GroEL^D473C/532Δ^ hetero-oligomers (Fig. 5), we investigated domain (Fig. 6A and B) incompatibilities that could be responsible for the lack of GroEL hetero-oligomer activity *in vivo*. GroEL chimeras consisting of E. coli/P. aeruginosa or E. coli/E. faecium domain swaps were generated and tested for their ability to rescue LG6 (Fig. 6). Chimeric *groEL* was QuickStep cloned (31) into *pBAD*-promoted plasmids with upstream E. coli *groES* present. Expression of P. aeruginosa GroEL with the equatorial domain replaced by E. coli rescued LG6, but not E. coli GroEL with the equatorial domain replaced by P. aeruginosa (Fig. 6C). E. faecium GroEL with E. coli equatorial and apical domains, but not E. coli equatorial domain replacement alone, was required for functional rescue of LG6 (Fig. 6C). These results suggest P. aeruginosa and E. faecium GroEL equatorial domains, in a mixed oligomer with E. coli GroEL, may disrupt positive and/or negative ring allostery and loss of chaperonin function. Furthermore, incompatibility of the E. faecium apical domain in the presence of E. coli GroES may contribute to the dominant-negative phenotype seen in Fig. 2.

### ESKAPE *groESL* knock-ins display similar growth kinetics and GroES/GroEL induction at various temperatures compared to the parent strain, but present with elongated phenotypes.

**FIG 7:**
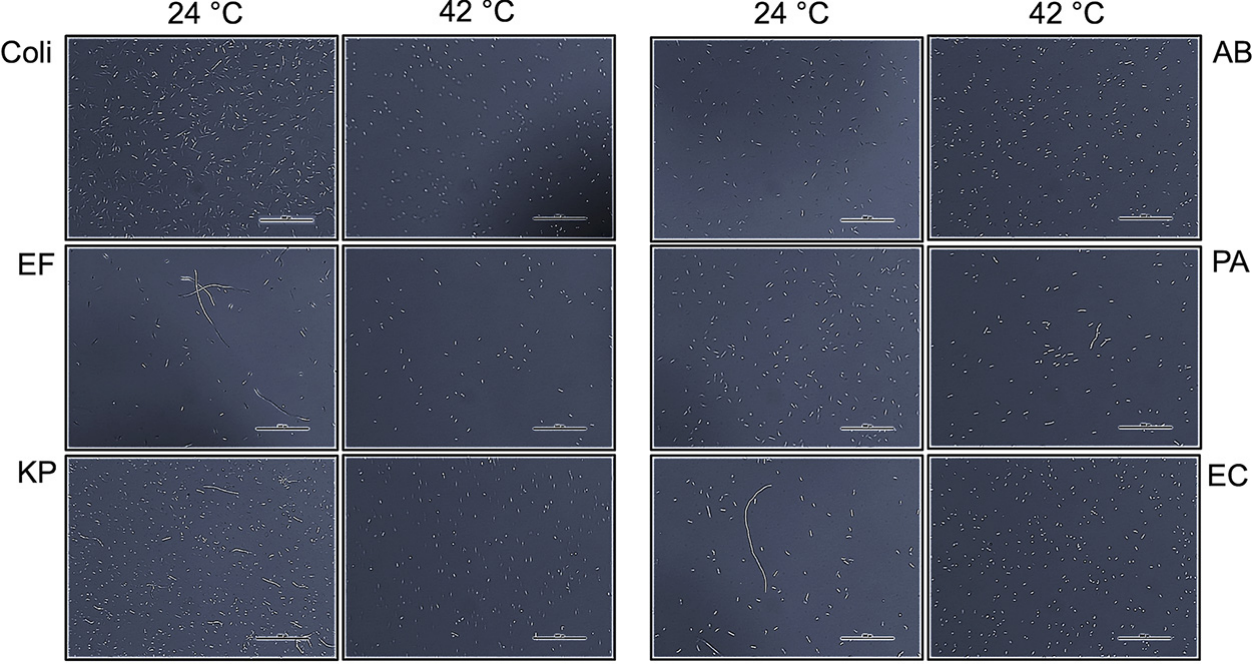
ESKAPE *groESL* knock-ins present with an elongated phenotype. MGΔ*groESL*::ESKAPE*groESL* (Cm^r^) cells show an elongated phenotype at various temperatures compared to the parent strain, MG1655, between 24 and 42°C. The 400× images were captured for each strain after growth to mid-log phase in LB medium without antibiotic at stated temperatures. Coli, E. coli MG1655 at 24°C (left) and 42°C (right); EF, MGΔ*groESL*::EF *groESL* at 24°C (left) and 42°C (right); KP, MGΔ*groESL*::KP *groESL* at 24°C (left) and 42°C (right); AB, MGΔ*groESL*::AB *groESL* at 24°C (left) and 42°C (right); PA, MGΔ*groESL*::PA *groESL* at 24°C (left) and 42°C (right); EC, MGΔ*groESL*::EC *groESL* at 24°C (left) and 42°C (right). The scale bar represents 80.5 μm.

**FIG 8:**
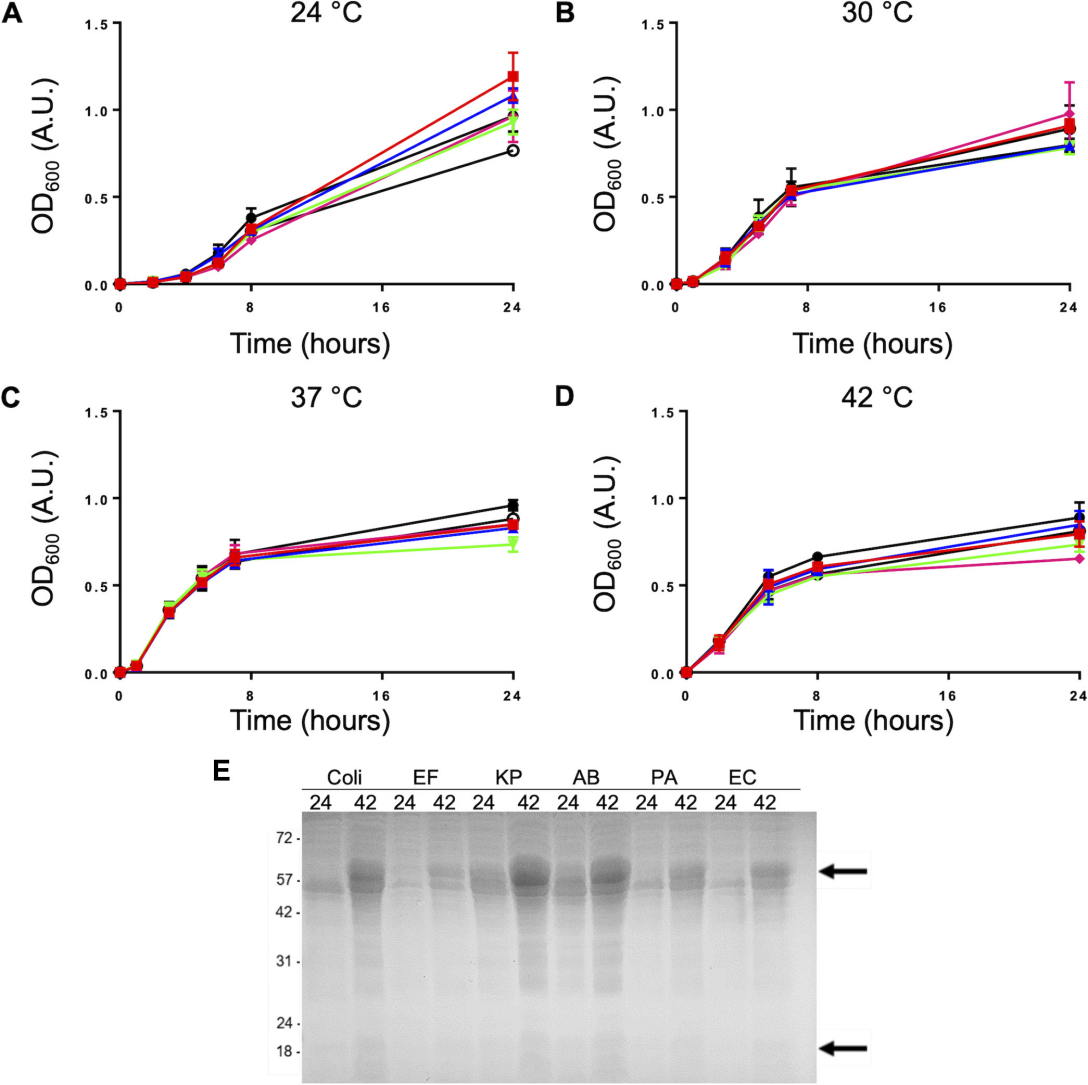
ESKAPE *groESL* knock-ins display similar growth kinetics and GroES/GroEL induction at various temperatures compared to the parent strain. MG1655Δ*groESL*::ESKAPE *groESL* (Cm^r^) shows similar growth kinetics and GroEL/ES induction compared to the parent strain, MG1655, between 24 and 42°C. In three independent experiments and reported as mean with SD, growth in LB medium without antibiotic at the stated temperature was measured by OD_600_ over time to determine growth rate of each individual strain. (A) Growth at 24°C. (B) Growth at 30°C. (C) Growth at 37°C. (D) Growth at 42°C. Black, MG1655; red, MGΔ*groESL*::EF *groESL* (Cm^r^); blue, MGΔ*groESL*::AB *groESL* (Cm^r^); green, MGΔ*groESL*::KP *groESL* (Cm^r^); pink, MGΔ*groESL*::PA *groESL* (Cm^r^); open/white, MGΔ*groESL*::EC *groESL* (Cm^r^). (E) Whole-cell lysates from E. coli and ESKAPE pathogens from MG1655 or knock-in strains expressing the respective GroES/GroEL were analyzed via SDS-PAGE. The black arrows indicate the positions of GroEL (upper) and GroES (lower). The lane numbers 24 and 42 represent 24 and 42°C for 5 min, respectively. Coli, E. coli; EF, MGΔ*groESL*::EF *groESL*; KP, MGΔ*groESL*::KP *groESL*; AB, MGΔ*groESL*::AB *groESL*; PA, MGΔ*groESL*::PA *groESL*; EC, MGΔ*groESL*::EC *groESL*.

The MG1655 wild-type strain and ESKAPE *groESL* knock-in strains were grown to mid-log phase at 24, 30, 37, or 42°C (for clarity, we only show data for 24 and 42°C) and imaged by bright-field microscopy at a total magnification of 400×. MG1655 and A. baumannii *groESL* knock-in strains did not display phenotypic abnormalities (Fig. 7). However, E. faecium, K. pneumoniae, and E. cloacae *groESL* knock-in strains displayed elongated phenotypes at 24°C, but not 42°C (Fig. 7), which is indicative of compromised GroEL function since GroEL is required for FtsZ function (7). P. aeruginosa *groESL* knock-ins displayed a normal phenotype at 24°C, but mild elongation compared to other strains at 42°C (Fig. 7). Despite these morphological changes at various temperatures, the growth rates of the ESKAPE *groESL* knock-in strains compared to the wild-type strain over 24 h appear unaffected from 24 to 42°C (Fig. 8A to D). To test the *groESL* operon response to heat stress, wild-type and ESKAPE knock-in strain GroES/GroEL induction at 24°C was compared to that in cells grown at 24°C and shifted to 42°C for 5 min. All strains were found to have heat-inducible GroES/GroEL as measured by SDS-PAGE, indicating preservation of functional *groE* operons (Fig. 8E). GroES/GroEL levels at 24 or 42°C were not found to be correlated with growth rate or phenotypic changes noted in Fig. 7

## DISCUSSION

**FIG 9:**
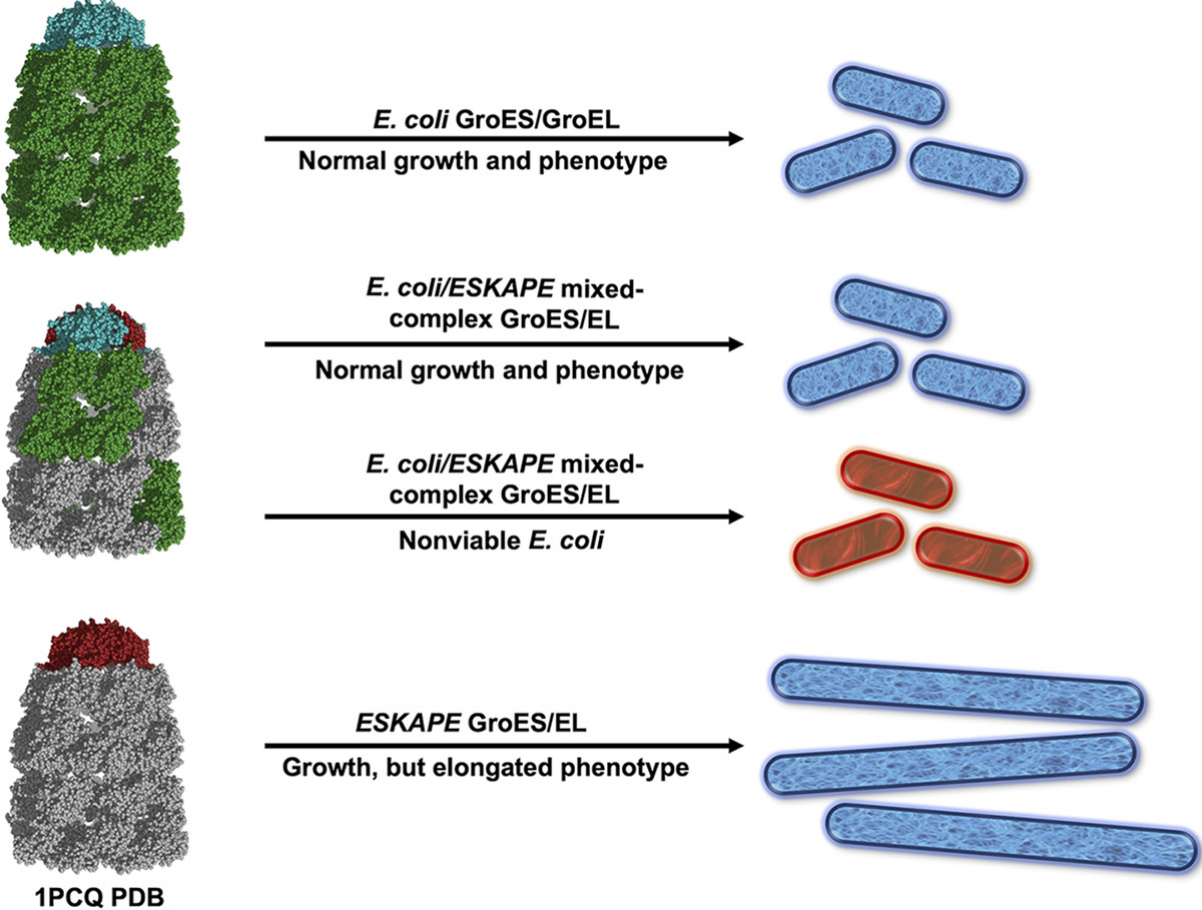
Dominant-negative phenotypes were observed either from hetero-oligomeric E. coli/ESKAPE GroEL or hetero-oligomeric GroES and GroEL, but complete replacement of E. coli *groESL* with ESKAPE *groESL* restored the organism’s viability and resulted in an elongated phenotype. The overall model is presented, including GroES/GroEL (PDB 1PCQ) showing E. coli GroES/GroEL (teal/forest green), ESKAPE GroES/GroEL (brick red/gray), and hetero-oligomeric GroES/GroEL (teal and brick red, forest green and gray) and viable (blue) or nonviable (red) E. coli cells with a normal or elongated phenotype.

Previous studies that replaced E. coli GroES/GroEL with homologs such as Cpn10/Cpn60 from Rhizobium leguminosarum or human mitochondrial Hsp10/Hsp60 have generated viable E. coli (32, 33). This type of complementation lends to the idea that chaperonin amino acid conservation between species parallels with similar client scopes. Therefore, it is not surprising that the intrinsic refolding actions among these systems are sufficient to sustain other organisms in some cases. Although complete replacement of E. coli GroES/GroEL with the chaperonins from other organisms is possible (33, 34), experiments where exogenous chaperonins were used to rescue GroES/GroEL-deficient E. coli, LG6 (18), have produced unexpected results (35–38). The most intriguing example came from the Mande group, where they studied the ability of Mycobacterium tuberculosis GroEL2 and E. coli/M. tuberculosis GroEL chimeras to rescue GroES/GroEL-deficient E. coli (39). Although M. tuberculosis GroEL2 is essential for M. tuberculosis survival, this chaperonin could not rescue GroES/GroEL-deficient E. coli despite significant amino acid identity with that of E. coli GroEL. Several chimeras that were generated in these studies existed as tetradecamers and could prevent aggregation of citrate synthase, but they could not rescue GroES/GroEL-deficient E. coli. This suggests that the GroEL chimeras were assembled as nonfunctional tetradecamers capable of trapping denatured protein, but not able to refold clients *in vivo*. We hypothesized that expression of GroEL^Coli^ and GroEL^ESKAPE^ in E. coli could lead to the generation of GroEL tetradecamers containing a mixture of GroEL^Coli^ and GroEL^ESKAPE^ subunits (Fig. 9). Because the refolding cycle of GroEL is dependent on the highly coordinated movement of multiple domains synchronized between subunits, positive allostery within the ring and/or negative allostery between the rings (40) could be altered by incorporation of dissimilar GroEL subunits. Some of these mixed tetradecamers may be hypofunctional or nonfunctional, perhaps capable of trapping misfolded protein, but unable to refold misfolded proteins *in vivo*. Organisms with multiple HSP60 isoforms seem to have evolved a mechanism to prevent mixture of endogenous HSP60s. Several groups have probed this and found that mixed endogenous oligomers were present at undetectable or very low levels (9, 41, 42); however, exceptions do exist (43, 44). Despite this, coexpression of GroEL^Coli^ and GroEL^Coli-mutant^ subunits can produce mixed GroEL tetradecamers and have been used to study E. coli GroEL function (29, 30).

E. coli strains were generated in which E. coli *groESL* was replaced by ESKAPE pathogen *groESL* to compare the extent to which these conserved chaperonin systems could function in E. coli. We predicted that the modest differences in amino acid similarity, isoelectric point, and total residue number between ESKAPE and E. coli GroES/GroEL (Table S2) were unlikely to cause divergence in chaperonin function or client recognition. Therefore, it was reasonable to predict that this set of chaperonin systems could complement a GroES/GroEL-deficient E. coli cell line. We discovered that the expression of GroES/GroEL from E. faecium, S. aureus, and P. aeruginosa in GroES/GroEL-deficient E. coli LG6 produced a dominant-negative phenotype that was not the result of codon bias or inappropriate gene dosage (Fig. 2; Table S3). Conversely, three other Gram-negative pathogens were able to rescue this GroES/GroEL-deficient cell line, including A. baumannii, whose GroEL sequence is least like E. coli GroEL compared to the other Gram-negative pathogen GroEL. ESKAPE pathogen GroES/GroEL that were unable to rescue LG6 undermined cellular viability in ways other than lack of expression; this was evident when GroESL^Coli^ was expressed from the chromosome of LG6 in the presence of ESKAPE pathogen *groESL* plasmid (Fig. 2F). Expression of GroES/GroEL from the LG6 chromosome produced cellular rescue in the presence of E. coli, K. pneumoniae, *A. baumanii*, and E. cloacae *groESL* plasmids or empty vector. However, when E. faecium, S. aureus, or P. aeruginosa *groESL* plasmids were present, these still failed to rescue despite the expression of GroES/GroEL from the chromosome of LG6. Together, these observations indicate that GroESL^Coli^ and GroESL^ESKAPE^ were being translated within LG6. However, in the presence of E. faecium, S. aureus, or P. aeruginosa chaperonin systems, viable LG6 colonies were not observed.

We next set out to test if the formation of mixed-nonfunctional GroEL complexes was responsible for the observed dominant-negative effect in E. coli by removing GroEL^Coli^ from the background of E. coli strain AI90, which retains a chromosomal copy of *groES*, but not *groEL* (Fig. 3). Here, AI90 is maintained by a plasmid copy of *groESL*, which can be selected against in the presence of sucrose. The formation of mixed-GroEL complexes is not possible in this system due to negative selection of the E. coli *groESL* and positive selection for ESKAPE *groESL* plasmid. GroEL ring mixing at the level of translation would be eliminated due to the absence of *the* E. coli *groEL*. This hypothesis was strengthened by the fact that P. aeruginosa, which previously could not rescue E. coli, was now able to rescue. We attribute this change to the loss of GroEL ring mixing, which was likely responsible for the dominant-negative phenotype seen with the P. aeruginosa GroES/GroEL chaperone system when expressed in the presence of GroEL^Coli^ in LG6. This observation does not explain the lack of rescue for Gram-positive chaperonin systems from E. faecium and S. aureus. It is possible that the presence of E. coli GroES and E. faecium or S. aureus GroES may disrupt the efficient refolding of clients due to a perturbed GroES-GroEL interaction and/or GroES function.

Next, we completely removed the possibility of GroESL^Coli^ and GroESL^ESKAPE^ subunit mixing for both GroES and GroEL by replacing E. coli *groESL* with ESKAPE *groESL*, while maintaining the upstream and downstream components of the E. coli *groE* operon (Fig. 4). Along with the Gram-negative ESKAPE pathogen chaperonin systems, which were found to rescue in earlier experiments, Gram-positive E. faecium *groESL* knock-in was now able to rescue in the absence of GroESL^Coli^.

Previous work in E. coli has shown that coexpression of GroEL^Coli^ and GroEL^Coli-mutant^ produced mixed tetradecamers (29, 30). This observation, along with work from the Lund and Mande groups, inspired the hypothesis that the coexpression of GroESL^Coli^ and GroESL^ESKAPE^ could produce the same phenomenon. This mixture of subunits could operate with enough functionality to maintain viable E. coli within some chaperone systems, but not others. Formation of mixed-GroEL complexes between E. coli chaperonin and E. faecium, S. aureus, and P. aeruginosa chaperonins, respectively, may perturb positive allostery within the GroEL ring and/or negative allostery between GroEL rings such that efficient refolding of essential gene products is compromised. This scenario would ultimately lead to loss of cell viability (8). Coexpression of GroEL^ESKAPE^ and GroEL^D473C/532Δ^ demonstrated that tetradecameric GroEL hetero-oligomers were formed *in vivo* (Fig. 5). Furthermore, *P* aeruginosa-E. coli and E. faecium-E. coli hetero-oligomers were found to be essentially devoid of ATPase activity (Fig. 5G), supporting our hypothesis regarding the dominant-negative phenotypes seen in the LG6 rescue experiment (Fig. 2).

Incompatibilities between GroEL^ESKAPE^ and GroEL^Coli^ domains were determined by generating GroEL chimeras (with the plasmid containing E. coli *groES* upstream of chimeric *groEL*) and screening for the functional rescue of LG6. For P. aeruginosa GroEL, rescue was possible by replacing the equatorial domain with the E. coli equatorial domain. For GroEL^E. faecium^ to rescue LG6, it was required that both the equatorial and apical domains be replaced by the E. coli equatorial and apical domains (Fig. 6C). Cochaperonin specificity has been documented (45); therefore, it is possible that chaperoning ability is compromised if E. coli GroES cannot efficiently interact with the E. faecium apical domain. It is recognized that the lack of a traditional GGM repeat in the C-terminal tail of GroEL may contribute to premature client release and decreased rate of refolding (46–48); however, replacement of the GroEL^E. faecium^ equatorial domain with that of GroEL^Coli^ (which contains the C-terminal GGM repeat) did not aid in the rescue of AI90 chimeric GroEL^E. faecium^. Furthermore, a traditional C-terminal GGM repeat was not required for GroESL^E. faecium^ rescue of E. coli (Fig. 4).

Each of the *groESL* knock-in strains displayed phenotypic changes at various temperatures, except for A. baumannii (Fig. 7). However, basal levels of GroES/GroEL or heat stress induction of knock-in ESKAPE *groESL* did not appear to affect growth rate compared to wild-type (Fig. 8). Inefficient FtsZ refolding by the GroES/GroEL chaperonin system (7, 49) may be responsible for the phenotypic changes seen with some of the ESKAPE *groESL* knock-in strains. It remains to be determined if these GroESL^ESKAPE^ chaperonins have divergent client scopes that are specific to their respective hosts, and this opens the possibility that their structure, allostery, and/or refolding cycle rates may differ to accommodate their own proteome.

### Conclusion.

We herein report a stepwise approach to study the ability of GroESL^ESKAPE^ chaperonin systems to rescue chaperonin-deficient E. coli. We found that the coexpression of GroESL^Coli^ and GroESL^ESKAPE^ generates mixed-subunit oligomers, some of which are nonfunctional and affect organism survival. These results build upon previous attempts to study exogenous GroES/GroEL within E. coli where background GroESL^Coli^ was present. This work highlights the need to eliminate background GroES/GroEL from the host strain as a requisite for recombinant expression to further study exogenous chaperonin systems. Future efforts will involve characterization of ESKAPE GroES/GroEL using the ESKAPE *groESL* knock-in strains we have generated. We wish to determine if, despite high conservation between ESKAPE and E. coli GroES/GroEL, these chaperone systems have evolved to refold different scopes of clients. Furthermore, these strains can be used to express ESKAPE GroES/GroEL without interference from host strain GroES/GroEL. Additionally, elucidation of ESKAPE GroEL allostery, GroES-GroEL interactions, and GroES/GroEL structures will be pursued.

## MATERIALS AND METHODS

### Plasmids and strains.

*pBAD*-promoted ESKAPE and E. coli *groESL* plasmids were generated by polymerase incomplete primer extension (PIPE) cloning using pSpeedET as the vector component and genomic DNA from Enterococcus faecium ATCC 51559, Staphylococcus aureus ATCC 25923, Klebsiella pneumonia ATCC 700603, Acinetobacter baumannii ATCC 19606, Pseudomonas aeruginosa ATCC 47085, Enterobacter cloacae ATCC 13047, and MG1655 K-12 *groESL* as insert components. *pBAD*-promoted chimera plasmids included E. coli *groES* upstream of chimeric *groEL* and were generated by QuickStep cloning (31). *lacIq-Ptac* GroEL^D473C/532Δ^ mutagenesis was performed using the Naismith method (50). Plasmid transformation into LG6 (from Horwich lab) was done by incubation of cells with 100 ng of plasmid for 20 min on ice, followed by 45 s of heat shock at 42°C. Cells were immediately returned to wet ice and diluted with 1 ml SOB (super optimal broth) medium after 2 min of incubation. Transformants were shaken for 1 h at 37°C, with or without induction/suppression agents (arabinose/dextrose), and plated at multiple dilutions on separate agar-antibiotic ± 0.2% arabinose or 0.5% dextrose. This same procedure was used for AI90, with exception of addition of 5 to 10% sucrose to agar plates.

### Gene knock-in.

ESKAPE *groESL* was knocked-in to the MG1655 K-12 *groE* operon using modified Datsenko-Wanner protocol (27, 28). MG1655 K-12 cells transformed with λ-red pKD46 plasmid were grown to an optical density at 600 nm (OD_600_) of 0.2 prior to induction with 0.2% arabinose. Cells were made electrocompetent after growth to an OD_600_ of 0.35 to 0.4 using several washes with ice-cold water and 10% glycerol. ESKAPE *groESL* genes were individually cloned into pKIKOarsB using traditional methods. Insertion cassette PCR products (including 50-bp overhangs covering upstream and downstream of the MG1655 K-12 *groE* operon) were transformed into MG1655 K-12 λ-red cells by electroporation using Bio-Rad Gene Pulser Xcell with 0.2-cm Gene Pulser electroporation cuvettes (C= 25 μF; PC = 200 Ω; V = 2.5 kV). Cells were shaken at 37°C in SOB medium for 3 h and plated on agar-antibiotic. Colonies that arose were picked and grown in LB medium/antibiotic, lysed by boiling at 100°C for 10 min, and then used as the DNA template in a PCR mixture containing primers that flank the *groE* operon. PCR products of potential knock-in colonies were sent for sequence confirmation. The chromosomal antibiotic resistance marker was removed using FLP-recombinase.

### Microscopy.

Knock-in strains or MG1655 K-12 cells were grown to an OD_600_ of 0.6 in LB medium and prepared in triplicate to be imaged after growth at 24 or 42°C. Live cells were diluted and added to Fisherbrand microscope slides prior to imaging on a Nikon Eclipse 50i microscope at a total magnification of 400×. Image colors were modified in Microsoft PowerPoint.

### Bacterial growth rate.

Knock-in strains or MG1655 K-12 cells were grown overnight in LB medium and prepared in triplicate after being diluted to an OD_600_ of 0.050. Diluted samples were grown at 24, 30, 37, or 42°C, with OD_600_ measurements taken at various time points, after which a growth curve was generated using GraphPad Prism.

### Chromosomal GroESL expression.

ESKAPE knock-in strains or MG1655 K-12 cells were grown to an OD_600_ of 0.6 at 24°C and then subjected to 5 min of heat shock at 42°C or continued growth at 24°C. Cells were pelleted, and supernatant was obtained by lysis with radioimmunoprecipitation assay (RIPA) buffer containing Halt protease cocktail (Thermo) and 1 mM phenylmethylsulfonyl fluoride (PMSF; Sigma), followed by centrifugation at 22,500 × *g* for 30 min at 4°C. Supernatant was diluted with Laemmli buffer and heated to 100°C for 10 min, then loaded onto a 15% polyacrylamide denaturing gel and resolved by electrophoresis. GroES and GroEL protein bands were visualized after staining with Coomassie blue.

### ATPase activity.

The malachite green assay (51) was used to detect the presence of inorganic phosphate post-ATP hydrolysis by GroEL. GroEL (50 nM) and ATP (100 μM) were incubated at room temperature in reaction buffer (50 mM Tris [pH 7.4], 50 mM KCl, 10 mM MgCl_2_, 1 mM DTT), with 50-μl aliquots removed from reaction mixture and added to 100 μl of malachite green in a 96-well clear plate (Greiner 655101) read at various time points using a SpectraMax ID5 plate reader at 660 nm.

### GroEL purification.

*pBAD* GroEL^ESKAPE^ and *lacIq-Ptac* GroEL^D473C/532Δ^ were transformed into BL21 in a stepwise fashion and induced for 45 min at 37°C using 0.2% arabinose and 0.5 mM IPTG in LB medium. Mixed complexes were first purified by Q Sepharose FF chromatography and then loaded onto thiopropyl Sepharose 4B resin and eluted with increasing amounts of DTT. Expression of GroEL^ESKAPE^ was done by transformation of *pBAD* GroEL^ESKAPE^ into the respective knock-in strain (Fig. 4) and induced with 0.2% arabinose for 4 h at 37°C after the culture reached an OD_600_ of 0.6. GroEL^D473C/532Δ^ expression was done by transformation of *lacIq-Ptac* GroEL^D473C/532Δ^ into BL21 and induced with 0.5 mM IPTG for 4 h at 37°C after the culture reached an OD_600_ of 0.6. Purification was carried out by Q Sepharose FF and TPS chromatography.

### Native PAGE.

E. coli GroEL^D473C/532Δ^, GroEL^ESKAPE^, or E. coli GroEL^D473C/532Δ^ and GroEL^ESKAPE^ hetero-oligomers were run under nondenaturing conditions in 1× native sample buffer (3× 3 ml glycerol, 6.4 ml H_2_O, bromophenol blue, 0.6 ml 50× running buffer) over 10 h at 80 V in 1× native running buffer (50× 7.5 g tris-base, 36 g glycine, H_2_O to 250 ml) on a 4 to 10% native gel (37.5:1 acrylamide/bis, APS [ammonium persulfate], TEMED [*N*,*N*,*N*′,*N*′-tetramethylethylenediamine], Tris [pH 6.8 stacking, pH 8.8 resolving], H_2_O). Separate samples were also run on the same gel after 5 min of heat denaturation in native sample buffer including 2% SDS and 10% β-mercaptoethanol (BME). Gels were stained in Coomassie blue and visualized by white light transillumination.

### Data availability.

Supporting information associated with this article can be found in the online version, which includes E. coli and ESKAPE pathogen amino acid conservation as well as codon usage for ESKAPE *groESL*.
