# Supplementary material for: Functional Differences between E. coli and ESKAPE Pathogen GroES/GroEL
Source: mBio. 2021 Jan 12;12(1):e02167-20. doi: 10.1128/mBio.02167-20 (PMC7844535; doi:10.1128/mBio.02167-20)
Supplement: TABLE S1 [file mBio.02167-20-st001.pdf]

Functional differences between *E. coli* and *ESKAPE* pathogen GroES/GroEL

Jared Sivinski[a], Andrew J. Ambrose[a], Iliya Panfilenko[a], Christopher J. Zerio[a], Jason M. Machulis[a], Niloufar Mollasalehi[b,c,d], Lynn K. Kaneko[a], Mckayla Stevens[e], Anne-Marie Ray[e], Yangshin Park[e,f,g], Chunxiang Wu[e,f,g], Quyen Q. Hoang[e,f,g], Steven M. Johnson[e], Eli Chapman[a,*]

[a] The University of Arizona, College of Pharmacy, Department of Pharmacology and Toxicology, 1703 E. Mabel St., PO Box 210207, Tucson, AZ 85721

[b] Department of Chemistry and Biochemistry, University of Arizona, Tucson, AZ 85721-0088

[c] Center for Innovation in Brain Science, Tucson, AZ 85721

[d] Department of Pharmacology, College of Medicine, University of Arizona, Tucson, AZ 85724

[e] Indiana University School of Medicine, Department of Biochemistry and Molecular Biology, 635 Barnhill Dr., Indianapolis, IN 46202

[f] Stark Neurosciences Research Institute, Indiana University School of Medicine. 320 W. 15th Street, Suite 414, Indianapolis, IN 46202

[g] Department of Neurology, Indiana University School of Medicine. 635 Barnhill Drive, Indianapolis, IN 46202

*Corresponding author: chapman@pharmacy.arizona.edu

TABLE S1. E. coli shares high amino acid similarity with ESKAPE pathogens[a].

A

Bacterium	*Enterococcus faecium*	*Staphylococcus aureus*	*Klebsiella pneumoniae*	*Acinetobacter baumannii*	*Pseudomonas aeruginosa*	*Enterobacter cloacae*	*Escherichia coli*
Enterococcus faecium	-	83.5	75.5	76	76.1	75.5	75.2
Staphylococcus aureus	83.5	-	74.5	75.5	74.3	74.2	74
Klebsiella pneumoniae	75.5	74.5	-	85.6	87.4	99.5	98
Acinetobacter baumannii	76	75.5	85.6	-	88.1	85.6	85.4
Pseudomonas aeruginosa	76.1	74.3	87.4	88.1	-	87.3	87.3
Enterobacter cloacae	75.5	74.2	99.5	85.6	87.3	-	98
Escherichia coli	75.2	74	98	85.4	87.3	98	-

B

Bacterium	*Enterococcus faecium*	*Staphylococcus aureus*	*Klebsiella pneumoniae*	*Acinetobacter baumannii*	*Pseudomonas aeruginosa*	*Enterobacter cloacae*	*Escherichia coli*
Enterococcus faecium	-	75.3	62.9	66	70.1	62.9	62.9
Staphylococcus aureus	75.3	-	66	67	66	63.9	62.9
Klebsiella pneumoniae	62.9	66	-	77.6	76.3	100	97.9
Acinetobacter baumannii	66	67	77.6	-	86.7	77.6	78.6
Pseudomonas aeruginosa	70.1	66	76.3	86.7	-	77.3	78.4
Enterobacter cloacae	62.9	63.9	100	77.6	77.3	-	97.9
Escherichia coli	62.9	62.9	97.9	78.6	78.4	97.9	-

C

Bacterium	*Enterococcus faecium*	*Staphylococcus aureus*	*Klebsiella pneumoniae*	*Acinetobacter baumannii*	*Pseudomonas aeruginosa*	*Enterobacter cloacae*	*Escherichia coli*
Enterococcus faecium	-	84.9	77.7	77.8	77.1	77.7	77.4
Staphylococcus aureus	84.9	-	75.8	77	75.8	75.8	75.8
Klebsiella pneumoniae	77.7	75.8	-	87	89.4	99.5	98
Acinetobacter baumannii	77.8	77	87	-	88.3	87	86.7
Pseudomonas aeruginosa	77.1	75.8	89.4	88.3	-	89.1	88.9
Enterobacter cloacae	77.7	75.8	99.5	87	89.1	-	98
Escherichia coli	77.4	75.8	98	86.7	88.9	98	-

[a]GroESL protein similarity (%) generated from EMBOSS Needle protein alignment of *E. coli* GroESL and ESKAPE pathogens. (A) GroESL protein similarity (%). (B) GroES protein similarity (%). (C) GroEL protein similarity (%). Color gradient demonstrates highest similarity highlighted in red, lowest similarity in white.
