# Supplementary material for: Functional Differences between E. coli and ESKAPE Pathogen GroES/GroEL
Source: mBio. 2021 Jan 12;12(1):e02167-20. doi: 10.1128/mBio.02167-20 (PMC7844535; doi:10.1128/mBio.02167-20)

Functional differences between *E. coli* and *ESKAPE* pathogen GroES/GroEL

Jared Sivinski[a], Andrew J. Ambrose[a], Iliya Panfilenko[a], Christopher J. Zerio[a], Jason M. Machulis[a], Niloufar Mollasalehi[b,c,d], Lynn K. Kaneko[a], Mckayla Stevens[e], Anne-Marie Ray[e], Yangshin Park[e,f,g], Chunxiang Wu[e,f,g], Quyen Q. Hoang[e,f,g], Steven M. Johnson[e], Eli Chapman[a,*]

[a] The University of Arizona, College of Pharmacy, Department of Pharmacology and Toxicology, 1703 E. Mabel St., PO Box 210207, Tucson, AZ 85721

[b] Department of Chemistry and Biochemistry, University of Arizona, Tucson, AZ 85721-0088

[c] Center for Innovation in Brain Science, Tucson, AZ 85721

[d] Department of Pharmacology, College of Medicine, University of Arizona, Tucson, AZ 85724

[e] Indiana University School of Medicine, Department of Biochemistry and Molecular Biology, 635 Barnhill Dr., Indianapolis, IN 46202

[f] Stark Neurosciences Research Institute, Indiana University School of Medicine. 320 W. 15th Street, Suite 414, Indianapolis, IN 46202

[g] Department of Neurology, Indiana University School of Medicine. 635 Barnhill Drive, Indianapolis, IN 46202

*Corresponding author: chapman@pharmacy.arizona.edu

Figure S1. Comparisons of the GroEL sequences from *E. coli* and the *ESKAPE* pathogens. The respective panels show GroEL sequence comparisons between *E. coli* 4pko.1.B with: **(EF)** *E. faecium*; **(SA)** *S. aureus*; **(KP)** *K. pneumoniae*; **(AB)** *A. baumannii*; **(PA)** *P. aeruginosa*; **(EC)** *E. cloacae*. Identical residues are shown in green, similar residues are shown in purple, and different residues are shown in red.

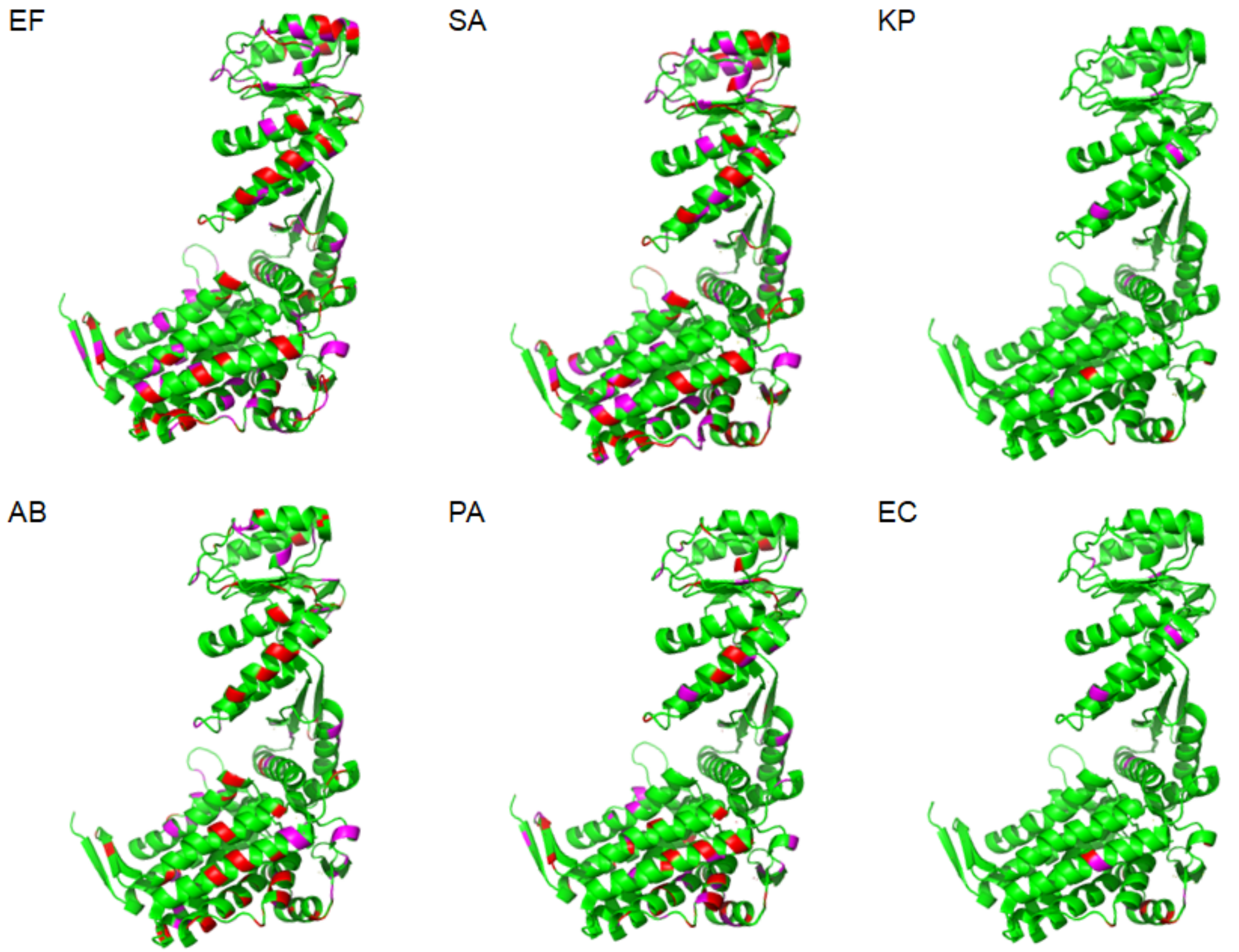

Supplement: FIG S1 [file mBio.02167-20-sf001.pdf]
