# Supplementary material for: Functional Differences between E. coli and ESKAPE Pathogen GroES/GroEL
Source: mBio. 2021 Jan 12;12(1):e02167-20. doi: 10.1128/mBio.02167-20 (PMC7844535; doi:10.1128/mBio.02167-20)
Supplement: TABLE S2 [file mBio.02167-20-st002.pdf]

Functional differences between *E. coli* and *ESKAPE* pathogen GroES/GroEL

Jared Sivinski[a], Andrew J. Ambrose[a], Iliya Panfilenko[a], Christopher J. Zerio[a], Jason M. Machulis[a], Niloufar Mollasalehi[b,c,d], Lynn K. Kaneko[a], Mckayla Stevens[e], Anne-Marie Ray[e], Yangshin Park[e,f,g], Chunxiang Wu[e,f,g], Quyen Q. Hoang[e,f,g], Steven M. Johnson[e], Eli Chapman[a,*]

[a] The University of Arizona, College of Pharmacy, Department of Pharmacology and Toxicology, 1703 E. Mabel St., PO Box 210207, Tucson, AZ 85721

[b] Department of Chemistry and Biochemistry, University of Arizona, Tucson, AZ 85721-0088

[c] Center for Innovation in Brain Science, Tucson, AZ 85721

[d] Department of Pharmacology, College of Medicine, University of Arizona, Tucson, AZ 85724

[e] Indiana University School of Medicine, Department of Biochemistry and Molecular Biology, 635 Barnhill Dr., Indianapolis, IN 46202

[f] Stark Neurosciences Research Institute, Indiana University School of Medicine. 320 W. 15th Street, Suite 414, Indianapolis, IN 46202

[g] Department of Neurology, Indiana University School of Medicine. 635 Barnhill Drive, Indianapolis, IN 46202

*Corresponding author: chapman@pharmacy.arizona.edu

TABLE S2. *ESKAPE* pathogen GroES/GroEL is predicted to be structurally similar with comparable net charge in their respective cellular environments as compared to MG1655 GroES/GroEL[a].

Bacteria	Gram Stain	GroES kDa/Residues	GroES PI	GroEL kDa/Residues	GroEL PI
Enterococcus faecium **ATCC 51559**	(+)	10.0 / 94	5.03	57.2 / 541	4.66
Staphylococcus aureus **ATCC 25923**	(+)	10.4 / 94	4.87	57.6 / 538	4.56
Klebsiella pneumonia **ATCC 700603**	(-)	10.4 / 97	5.38	57.1 / 548	4.84
Acinetobacter baumannii **ATCC 19606**	(-)	10.1 / 96	5.09	57.1 / 544	4.92
Pseudomonas aeruginosa **ATCC 47085**	(-)	10.3 / 97	5.17	57.1 / 547	5.04
Enterobacter cloacae **ATCC 13047**	(-)	10.4 / 97	5.38	57.1 / 548	4.85
Escherichia coli **LG6**	(-)	10.4 / 97	5.15	57.3 / 548	4.85

[a]Isoelectric point and molecular weight data were generated using ExPASy Computer pI/MW data tool. Gram-positives (*E. faecium* and *S. aureus*) have overall fewer number of residues, lower isoelectric point, and are lacking C-terminal GGM repeat compared to Gram-negative bacteria (*K. pneumonia*, *A. baumannii*, *P. aeruginosa*, *E. cloacae*, and *E. coli)*.
