# Supplementary material for: Functional Differences between E. coli and ESKAPE Pathogen GroES/GroEL
Source: mBio. 2021 Jan 12;12(1):e02167-20. doi: 10.1128/mBio.02167-20 (PMC7844535; doi:10.1128/mBio.02167-20)
Supplement: TABLE S3 [file mBio.02167-20-st003.pdf]

Functional differences between *E. coli* and *ESKAPE* pathogen GroES/GroEL

Jared Sivinski[a], Andrew J. Ambrose[a], Iliya Panfilenko[a], Christopher J. Zerio[a], Jason M. Machulis[a], Niloufar Mollasalehi[b,c,d], Lynn K. Kaneko[a], Mckayla Stevens[e], Anne-Marie Ray[e], Yangshin Park[e,f,g], Chunxiang Wu[e,f,g], Quyen Q. Hoang[e,f,g], Steven M. Johnson[e], Eli Chapman[a,*]

[a] The University of Arizona, College of Pharmacy, Department of Pharmacology and Toxicology, 1703 E. Mabel St., PO Box 210207, Tucson, AZ 85721

[b] Department of Chemistry and Biochemistry, University of Arizona, Tucson, AZ 85721-0088

[c] Center for Innovation in Brain Science, Tucson, AZ 85721

[d] Department of Pharmacology, College of Medicine, University of Arizona, Tucson, AZ 85724

[e] Indiana University School of Medicine, Department of Biochemistry and Molecular Biology, 635 Barnhill Dr., Indianapolis, IN 46202

[f] Stark Neurosciences Research Institute, Indiana University School of Medicine. 320 W. 15th Street, Suite 414, Indianapolis, IN 46202

[g] Department of Neurology, Indiana University School of Medicine. 635 Barnhill Drive, Indianapolis, IN 46202

*Corresponding author: chapman@pharmacy.arizona.edu

Table S3. *ESKAPE* pathogen GroES/GroEL require less than 3% *E. coli* rare codons.

	CODONS	TTT	TTC	TTA	TTG	CTT	CTC	CTA	CTG	ATT	ATC	ATA	GTT	GTC	GTA	GTG
	AMINOACIDS	F	F	L	L	L	L	L	L	I	I	I	V	V	V	V
EF		6.279	6.279	32.967	32.967	4.71	0	10.989	4.71	23.548	45.526	0	43.956	15.699	40.816	10.989
SA		7.886	7.886	56.782	7.886	6.309	0	7.886	0	47.319	17.35	0	56.782	6.309	31.546	15.773
KP		1.546	12.365	3.091	0	0	1.546	0	68.006	6.182	58.733	0	46.368	12.365	20.093	34.003
AB		7.788	9.346	34.268	6.231	29.595	1.558	0	0	34.268	42.056	0	56.075	0	40.498	6.231
PA		0	12.384	0	0	1.548	4.644	0	77.399	1.548	55.728	0	15.48	47.988	10.836	41.796
EC		0	13.91	3.091	1.546	0	1.546	0	64.915	10.819	54.096	0	44.822	12.365	21.638	34.003
Coli		0	12.365	1.546	4.637	1.546	1.546	0	66.461	9.274	55.641	0	54.096	6.182	21.638	27.821

	CODONS	TCT	TCC	TCA	TCG	AGT	AGC	CCT	CCC	CCA	CCG	ACT	ACC	ACA	ACG	GCT
	AMINOACIDS	S	S	S	S	S	S	P	P	P	P	T	T	T	T	A
EF		18.838	1.57	15.699	1.57	0	1.57	7.849	1.57	18.838	0	18.838	0	40.816	6.279	45.526
SA		14.196	0	12.618	0	11.041	3.155	9.464	0	15.773	1.577	25.237	0	34.7	7.886	26.814
KP		13.91	17.002	1.546	0	1.546	3.091	1.546	0	1.546	23.184	7.728	46.368	0	1.546	43.277
AB		23.364	1.558	14.019	0	0	4.673	4.673	0	17.134	4.673	43.614	1.558	14.019	0	66.978
PA		0	26.316	0	3.096	0	9.288	4.644	4.644	0	21.672	3.096	41.796	0	0	26.316
EC		13.91	20.093	1.546	1.546	1.546	3.091	6.182	0	13.91	6.182	10.819	40.185	0	4.637	41.731
Coli		21.638	10.819	0	0	0	3.091	1.546	0	6.182	17.002	13.91	41.731	0	0	55.641

	CODONS	GCC	GCA	GCG	TAT	TAC	CAT	CAC	CAA	CAG	AAT	AAC	AAA	AAG	GAT	GAC
	AMINOACIDS	A	A	A	Y	Y	H	H	Q	Q	N	N	K	K	D	D
EF		10.989	58.085	3.14	6.279	6.279	4.71	0	21.978	1.57	15.699	23.548	72.214	3.14	40.816	18.838
SA		0	59.937	11.041	11.041	1.577	3.155	0	41.009	0	39.432	15.773	61.514	3.155	47.319	14.196
KP		9.274	34.003	40.185	1.546	10.819	1.546	1.546	0	23.184	1.546	34.003	66.461	9.274	15.456	49.459
AB		0	42.056	10.903	6.231	3.115	1.558	6.231	24.922	1.558	12.461	28.037	60.748	7.788	28.037	34.268
PA		88.235	4.644	7.74	0	9.288	1.548	3.096	4.644	20.124	0	35.604	15.48	58.824	13.932	41.796
EC		10.819	26.275	46.368	1.546	10.819	1.546	1.546	1.546	21.638	3.091	30.912	63.369	12.365	15.456	46.368
Coli		4.637	41.731	23.184	0	12.365	1.546	1.546	0	24.73	7.728	29.366	64.915	9.274	20.093	43.277

	CODONS	GAA	GAG	TGT	TGC	CGT	CGC	CGA	CGG	AGA	AGG	GGT	GGC	GGA	GGG	ATG	TGG
	AMINOACIDS	E	E	C	C	R	R	R	R	R	R	G	G	G	G	M	W
EF		95.761	3.14	0	0	18.838	12.559	1.57	0	0	0	47.096	12.559	23.548	6.279	28.257	1.57
SA		96.215	6.309	0	0	26.814	9.464	3.155	0	1.577	0	52.05	15.773	11.041	3.155	28.391	1.577
KP		71.097	12.365	0	3.091	26.275	13.91	0	0	0	0	35.549	69.552	1.546	1.546	37.094	0
AB		76.324	6.231	3.115	0	28.037	14.019	0	0	0	0	76.324	23.364	1.558	1.558	34.268	0
PA		49.536	38.7	0	1.548	15.48	27.864	0	0	0	0	20.124	88.235	0	1.548	38.7	0
EC		71.097	15.456	0	3.091	29.366	10.819	0	0	0	0	54.096	52.55	1.546	0	37.094	0
Coli		80.371	6.182	0	4.637	30.912	10.819	0	0	0	0	44.822	61.824	0	0	38.64	0

CAIcal codon usage per 1000 bp *ESKAPE* and *E. coli groESL*. *E. coli* rare codons are highlighted in red with relative codon usage per 1000 bp quantified in blue. *E. faecium* (EF); *S. aureus* (SA); *K. pneumoniae* (KP); *A. baumannii* (AB); *P. aeruginosa* (PA); *E. cloacae* (EC); *E. coli* (Coli).
